# Elevated CAIX Expression is Associated with an Increased Risk of Distant Failure in Early-Stage Cervical Cancer

**DOI:** 10.4137/bmi.s570

**Published:** 2008-02-01

**Authors:** John P. Kirkpatrick, Zahid N. Rabbani, Rex C. Bentley, Matt E. Hardee, Seth Karol, Jeffrey Meyer, Egbert Oosterwijk, Laura Havrilesky, Angeles Alvarez Secord, Zeljko Vujaskovic, Mark W. Dewhirst, Ellen L. Jones

**Affiliations:** 1 Department of Radiation Oncology, Duke University Medical Center, Durham, NC, U.S.A. 27710; 2 Department of Pathology, Duke University Medical Center, Durham, NC, U.S.A. 27710; 3 Department of Experimental Urology, University of Nijmegen, Nijmegen, Netherlands; 4 Department of Obstetrics-Gynecology, Duke University Medical Center, Durham, NC, U.S.A. 27710

**Keywords:** tumour hypoxia, carbonic anhydrase IX, uterine cervical cancer

## Abstract

Tumor hypoxia is associated with adverse outcome in many malignancies. The goal of this study was to determine if elevated expression of carbonic anhydrase IX (CAIX), a biomarker of hypoxia, predicts for recurrence in early-stage cervical cancer. The charts of all patients with early-stage cervical cancer, primarily FIGO IB, treated by radical hysterectomy at our institution from 1988–2001 were reviewed. Adequate pathologic specimens from patients who recurred or who had at least three years follow-up and remained disease-free were stained for CAIX. An immunohistochemical score (IHC) was generated from the extent/intensity of staining. Outcome, as measured by freedom from recurrence (FFR), distant metastases (FFDM) and local recurrence (FFLR), was analyzed as a function of age, IHC, lymph node status (LN) and histology. Forty-two relapsing patients and 76 non-relapsing patients were evaluated. In univariate analysis, +LN, though not IHC or histology, was a significant predictor of any recurrence. Both +LN and higher IHC were associated with decreased FFDM but not FFLR. Patients with both +LN and elevated IHC more frequently exhibited distant metastases as first site of failure (5-year FFDM 50%) than patients with only +LN, elevated IHC or neither feature (70, 85 and 95%, respectively, p = 0.0004). In multivariable analysis, only +LN was significantly associated with poorer FFDM (hazard ratio 4.6, p = 0.0015) though there was a strong trend with elevated CAIX expression (p = 0.069). Elevated CAIX expression is associated with more frequent distant metastases in early-stage cervical cancer, suggesting that patients with this characteristic may benefit from more aggressive treatment.

## Introduction

In a variety of human cancers, including soft tissue sarcomas, head-and-neck squamous cell carcinomas and cancer of the uterine cervix, tumor hypoxia is associated with an increased incidence of metastases, decreased progression-free survival and poor outcome (Dische et al. 1983; Fein et al. 1995; Höckel et al. 1996 and 1999; Brizel et al. 1996 and 1997; Nordsmark et al. 1996; Wykoff et al. 2000; Vordermark and Brown, 2003; Kim et al. 2005). As a marker for unfavorable prognosis, hypoxia has been targeted in a variety of ways and is the subject of active ongoing clinical research. This includes a phase III trial for patients with cervical cancer receiving chemoradiation who are randomized to standard therapy with or without tirapazamine, a hypoxic cell sensitizer (GOG 0219). To date, clinical efforts have primarily focused on the role of hypoxia in locally advanced cancers. However, there are subsets of early-stage cancer patients who have unexpectedly poor clinical outcomes. This retrospective study focuses on a large group of patients with early-stage cervical cancer treated definitively with radical surgery.

Tumor hypoxia (PO_2_) has been shown to promote the selection of cells with reduced apoptotic potential (Graeber et al. 1996), to increase the frequency of mutations (Reynolds et al. 1996; Sutherland, 1998), to induce genes promoting angiogenesis (Dachs and Chaplin, 1998) and to decrease radiosensitivity (Gray et al. 1953; Bedford and Mitchell, 1974). Endogenous hypoxia biomarkers are genes or gene products specifically up-regulated under hypoxic conditions. Candidate biomarkers have included hypoxia-inducible factor 1-α (HIF-1α) and its downstream products, vascular endothelial growth actor (VEGF) and metallomatrixproteinases (Kim et al. 2005; Moon et al. 2007; Harris, 2002). Carbonic anhydrase 9 (CA9, Opavsky et al. 1996) is a HIF-1 target gene (Wykoff et al. 2000) whose product, carbonic anhydrase IX (CAIX), has been investigated as an endogenous hypoxia biomarker (Olive et al. 2001). CAIX is a transmembrane protein that catalyzes the reversible hydration of carbon dioxide to carbonic acid, regulating intracellular pH (Ivanov et al. 1998). Thus, CAIX appears to help maintain a normal pH in tumor cells under hypoxic conditions, allowing tumors to acclimate to a hostile microenvironment. In turn, this facilitates tumor proliferation in hypoxic regions (Blancher and Harris, 1998; Yamagata et al. 1998).

We hypothesized that tumor hypoxia might be associated with an inherently more malignant phenotype expressed at a relatively early point in the evolution of these tumors. This retrospective study explores the association between CAIX expression, other tumor/patient factors and outcome in early-stage cervical cancer (primarily FIGO IB) treated definitively by radical hysterectomy.

## Materials and Methods

### Patients

This was a single-institution retrospective study, approved by the Institutional Review Board. We reviewed the charts of all women who underwent a radical hysterectomy (RH) at Duke University Medical Center as definitive treatment for early-stage cervical cancer between 1988 and 2001. A total of 315 patients, predominantly FIGO stage IB, were identified. Pathologic specimens from any patient who either recurred (96 patients) *or* who had a follow-up of at least three years and remained disease-free (49 patients) were examined by a pathologist blinded to outcome (RCB). In turn, those blocks with adequate tumor specimens (118 total patients) were cut and the slides stained for carbonic anhydrase IX (CAIX).

### CAIX staining

Immunohistochemistry was performed using 5-μm sections of formalin-fixed, paraffin-embedded tissues placed onto positively charged glass slides, followed by routine dewaxing and rehydration. Negative controls were included by omission of the primary antibody. CAIX expression was detected using anti-CAIX mouse monoclonal antibody clone M75 (gift from E. Oosterwijk, University Hospital Nijmegen, Nijmegen, Netherlands). After peroxidase was quenched with methanol and 3% hydrogen peroxide for 10 minutes, microwave antigen retrieval was done twice on 600W for 5 minutes each. After blocking with 10% donkey serum, the slides were incubated with the primary antibody (3 μg/ml) overnight at 4 °C, and washed with Tris-buffered saline. Biotinylated donkey antimouse antibody (1:1000 volume/volume, Jackson ImmunoResearch, West Grove, PA) was applied for 30 minutes at room temperature, followed by application of ABC kit (Vector Lab, Inc., Burlingame, CA, USA).

**Figure 1:**
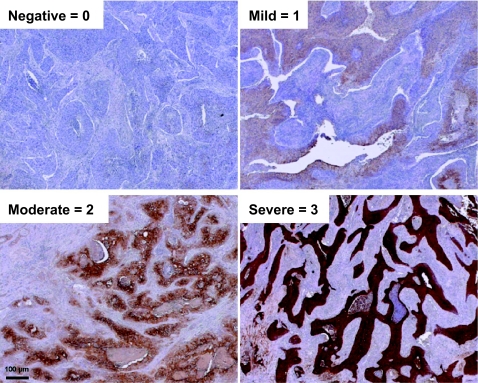
Examples of CAIX staining intensity of human cervical cancer: **a**. none = 0, **b**. mild = 1, **c**. moderate = 2, **d**. strong = 3.

Following staining, specimens were evaluated by two other pathologists (ZNR, MEH) blinded to the patient identity and clinical outcome. On each slide, the entire area containing tumor was examined under low power (40X). Each pathologist determined intensity (0 = no staining, 1 = mild staining, 2 = moderate staining, 3 = strong staining) and the extent of all tumor cells (0%–100%) with each intensity of staining, independently. Representative examples are shown in Figure 1. For the purposes of this analysis, only the product of the strong intensity (3) and the average extent of staining for this intensity was used to calculate the immunohistochemical (IHC) score for each specimen. A breakpoint of <30% versus ≥30% IHC score was selected for the outcome analyses, as the outcome of the groups falling above versus below this value appeared quite different and there was an adequate number of patients for statistical analysis within each group.

### Statistics

Survival curves were generated (Kaplan and Meier, 1958) and compared by the log-rank test (Mantel and Haenszel, 1958). The associations of patient/tumor characteristics with survival were determined by multivariable Cox proportional-hazards analyses (Cox, 1972). The principal endpoints examined were freedom from recurrence (FFR), freedom from distant metastases as the first site of recurrence (FFDM) and freedom from local failures as the first site of recurrence (FFLR). When recurrence was detected simultaneously at distant sites and locally, the patient was scored as having both a local recurrence and distant failure on that date. The multivariable proportional hazard model was built using forward stepwise selection. In building this model, the effect of the independent variables (age, lymph node status, IHC score and histology) were tested, as well as the cross products (e.g., lymph nodes status × IHC score) of these variables. All p-values reported were two-sided and statistical significance was defined as p < 0.05. Statistical analyses were performed using JMP software version 6 (SAS Institute, Inc., Cary, North Carolina).

## Results

**Figure 2:**
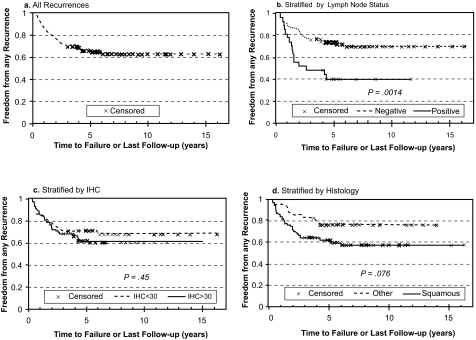
Probability of freedom from any recurrence (FFR) versus time after radical hysterectomy for all patients (**a**). and stratified by positive lymph node (LN) status (**b**., positive versus negative), immunohistochemical (IHC) score (**c**., <30 versus ≥30) and tumor histology (**d**., squamous cell carcinoma versus other).

Forty-two relapsing patients and 76 non-relapsing patients with a minimum of 3 years follow-up had adequate pathology specimens and were included in the analysis (Table 1.) In the overall group of 118 patients, the median follow-up was 59.6 months (range 4–195 months), the median age at the time of surgery was 44.9 years (range 19.4–79.8 years) and 27 patients were found to have positive pelvic and/or para-aortic lymph nodes at the time of surgery. The histology was squamous cell carcinoma, adenocarcinoma, adenosquamous carcinoma and other in 84, 22, 7 and 5 patients, respectively. FIGO stage was IA, IB and IIA in 3, 105, and 5 patients; it could not be determined definitively from the clinical data in 5 patients. The two groups did not differ significantly in age, with the median age 44.3 and 45.1 years in the relapsing and non-relapsing patients, respectively. Of the 42 relapsing patients, the first site(s) of failure was local in 32 and distant in 19 patients. For 9 patients, synchronous recurrent disease was first detected locally and at distant sites. In the overall group, freedom from any recurrence (FFR) at 3, 5 and 10 years was 69, 64 and 63%, respectively (Fig. 2). Positive lymph node status at surgery was associated with a significantly worse 5-year FFR, 40 versus 72% (p = 0.0013, Table 2).

**Figure 3:**
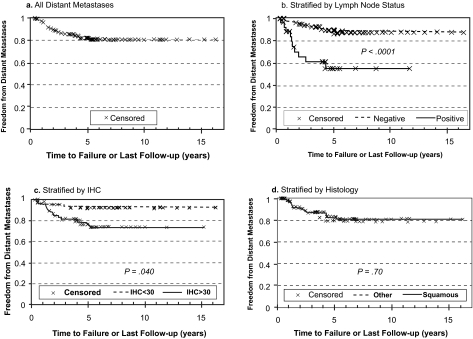
Probability of freedom from distant metastases (FFDM) as first site of recurrence versus time after radical hysterectomy for all patients (**a**). and stratified by lymph node (LN) status (**b**., positive versus negative), immunohistochemical (IHC) score (**c**., <30 versus ≥30) and tumor histology (**d**., squamous cell carcinoma versus other).

**Figure 4:**
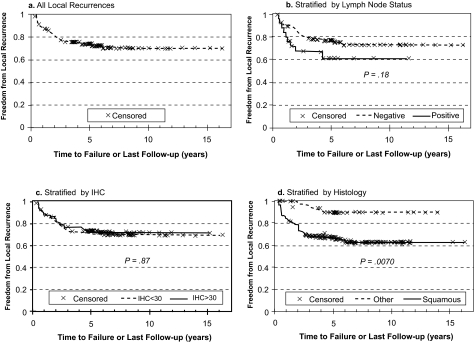
Probability of freedom from local recurrence (FFLR) as first site of recurrence versus time after radical hysterectomy for all patients (**a**). and stratified by lymph node (LN) status (**b**., positive versus negative), immunohistochemical (IHC) score (**c**., <30 versus ≥30) and tumor histology (**d**., squamous cell carcinoma versus other).

Though there was a weak trend for poorer FFR associated with higher IHC score and extent of CAIX staining, the difference was not significant. However, when FFR was stratified by distant metastases and local recurrence as the first site(s) of failure, significantly higher freedom from distant metastasis (FFDM) was associated with reduced CAIX staining (92 versus 74% at 5 years for low versus high IHC, p = 0.040) as shown in Figure 3. In contrast, local recurrence (Fig. 4). appeared to be independent of the level of CAIX expression (p = 0.87). Similarly, positive lymph node status at surgery was strongly associated with decreased FFDM (91 versus 55% at 5 years for negative versus positive lymph nodes, p < 0.0001), while there was no significant association with the nodal status and local recurrence. Conversely, there was a significant association between squamous histology and poorer freedom from local recurrence (FFLR 70% for squamous histology versus 90% for other histologies, p = 0.007, Figure 4) but no apparent association with distant metastases (Fig. 3).

**Figure 5:**
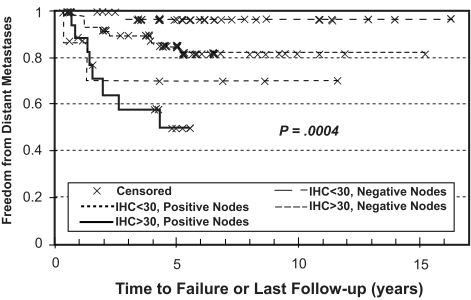
Probability of freedom from distant metastases (FFDM) as first site of recurrence versus time after radical hysterectomy stratified by negative lymph nodes (LN) and immunohistochemical score (IHC) <30 (n = 37), negative LN and IHC ≥30 (n = 54), positive LN and IHC <30 (n = 8) and positive LN and IHC ≥30 (n = 19.)

The results of the survival analysis for the combination of CAIX staining and nodal status are shown in Figure 5. The combination of a high CAIX IHC score and positive lymph nodes was associated with a significantly poorer FFR and FFDM but not FFLR. For patients with elevated CAIX IHC score and positive nodes, FFDM at 5 years was significantly lower (50%) than that of patients with either positive nodes or elevated CAIX score alone or with neither adverse feature (5-year FFDM 70, 85 or 95%, respectively, p = 0.0004.) Likewise, the 5-year FFR’s for these combination of features were 35, 50, 72 and 76%, respectively (p = 0.0021.) Thus, the finding of elevated CAIX expression is associated with an increased incidence of distant metastases, independent of lymph node status. These results are summarized in Table 3.

The results of the multivariable proportional hazards analysis, presented in Table 4, are consistent with those of the univariate analyses. In the 4-variable models based on age, lymph node status, IHC score and histology, age never reached the level of significance. In the 3-variable (lymph node status, IHC score and histology) and 2-variable (lymph node status and histology) models of FFR, both lymph node status and histology were significantly associated with FFR. While the 3-variable mode of local recurrence showed both histology and lymph node status to be significantly associated with FFLR, in the 2-variable model only histology was statistically significant. In contrast, in both the 3- and 2-variable models of FFDM, lymph node status was significantly associated with distant metastases as the first site of failure, while IHC score trended toward significance and histology had no apparent association. When cross products (IHC score × lymph node status, IHC score × histology, lymph node status × histology) were included in the 3-variable model for FFDM, both IHC score (HR = 4.99, p = 0.030) and lymph node status (HR = 6.76, p = 0.0015) were significant.

## Discussion

### Prognostic significance of CAIX overexpression

This study supports the hypothesis that hypoxia plays a key role in tumor metastasis, even for early-stage cancers. In several studies, CAIX expression appears closely associated with tumor hypoxia (Jankovic et al. 2006; Loncaster et al. 2001; Airley et al. 2003), while in others the relationship between CAIX expression and hypoxia is unclear (Hedley et al. 2003; Mayer et al. 2005). Likewise, the prognostic significance of CAIX is controversial. While some studies have revealed significant associations between CAIX and adverse outcome (Kim et al. 2005; Koukourakis et al. 2001 and 2006; Giatromanolaki et al. 2001; Swinson et al. 2003; Kim et al. 2006), others have shown no significant association (Kaanders et al. 2002; Hedley et al. 2003).

Our results for CAIX overexpression in early stage cervix cancer are consistent with those of Loncaster et al. (2001) who found a significantly worse metastasis-free and disease-specific survival but no difference in local control when CAIX was overexpressed. That study was based on a retrospective series of 130 locally advanced patients treated with definitive radiation therapy. Likewise, Kim et al. (2006) found that CAIX overexpression was the most statistically significant factor associated with distant metastases-free survival in a group of 59 primarily locally advanced cervical cancer patients treated with radiotherapy. However, their multivariable analysis did not include lymph node status as a one of the factors. In contrast, a series of 110 patients from Hedley et al. (2003) did not find significant associations between CAIX expression and either tumor pO_2_ levels or patient outcome in locally advanced carcinomas of the cervix treated with radiotherapy or radiochemotherapy. While this study looks at a patient population with an earlier stage of disease treated by a different modality (surgery, rather than radiotherapy), there appears to be a significant relationship between CAIX expression and increased metastatic potential. Thus, these results suggest that a hypoxic *micro*environment is directly associated with a more aggressive cervical cancer phenotype present early in development of a tumor and that the poorer outcome is not merely due to decreased radioresistance of the more hypoxic tumors.

The cause-and-effect relationship between tumor hypoxia, HIF-1α, CAIX and clinical outcome remains unsettled (Moon et al. 2007). For example, in a study of locally advanced cervical cancer, Hutchison et al. (2004) found weak, but statistically significant, correlations of HIF-1α expression with lower tumor pO_2_ and CAIX but no correlation of HIF-1α with clinical outcome. Elucidation of these relationships is complicated by the inherent macroscopic and microscopic heterogeneity of partially hypoxic tissues. Cline et al. (1997) have shown that sampling errors related to this heterogeneity can produce significant variations in the estimated fraction of hypoxic tissue. In addition, determining the extent and intensity of staining for an endogenous hypoxic marker is technique dependent. It is not clear how much of the apparent discrepancies in clinical outcome versus the extent/intensity of CAIX staining between studies is due to differences in staining methodology.

Other series have demonstrated associations between hypoxia and nodal status using radiologic assessment. Fyles et al. (2002) demonstrated hypoxia as an independent predictor of outcome only in patients with node negative cervix cancer, again using radiologic nodal assessment in the setting of definitive radiation therapy. The well-established association between positive lymph node status and adverse outcome (Sundfor et al. 1998; Ho et al. 2004) was also confirmed in our study.

### Measurement of tumor oxygenation

Typically, tumor oxygenation is directly measured using microelectrodes or RuO_2_ microprobes (Brown and Le, 2002). However, these techniques involve an invasive procedure, which restricts their use to accessible tumors. Hypoxia marker drugs, such as nitromidazole derivatives (e.g., EF5 and pimonidazole), also have been used to detect hypoxia in tissue specimens. Though these methods effectively and quantitatively identify the extent and degree of hypoxia, the requirement for administration prior to biopsy limits their clinical use to prospective studies (Kaanders et al. 2002; Evans et al. 2000). Alternatively, it would be advantageous to assess tumor hypoxia using endogenous markers that can be detected in routine clinical biopsy specimens, obviating the need for an invasive procedure and pre-biopsy marker drug administration (Moon et al. 2007). Thus, one potentially powerful advantage of the CAIX technique is that it can be applied to paraffin-embedded tissues, enabling analysis of relatively large series with long clinical follow-up.

### Clinical implications of tumor hypoxia

Tumor hypoxia is a complex entity which is a function of both oxygen supply and demand. On the supply side, deficiencies in the nutrient supply, such as low vascular density and uneven vascular distribution may play a role. On the demand side, abnormalities in energy metabolism are known to be related to defects in regulation of respiratory enzymes, combined with high demand for energy in the setting of rapid cellular proliferation. A shift toward anaerobic metabolism would decrease oxygen consumption rates, which could lead to improvement in tumor oxygenation. Theoretical modeling of the relative contributions from perfusion and consumption on tumor oxygenation came to the conclusion that manipulation of oxygen consumption is an order of magnitude more effective in changing tumor hypoxia than changing perfusion (Secomb et al. 1998). Thus, even a small decrease in respiration (i.e., oxygen consumption) could potentially lead to profound changes in tumor oxygenation.

Low oxygen tension appears to be a clinically relevant feature of the tumor microenvironment, but the optimal intervention to ameliorate the effects of hypoxia remains uncertain (Coleman et al. 2004). Possible avenues of exploration include strategies to correct the underlying pathophysiologic process which led to hypoxia, and, thus, to influence the downstream expression of angiogenic and apoptotic factors. Alternatively, the microenvironment could be left unperturbed and hypoxic cell sensitizers or hypoxic cytotoxic therapy, such as tirapazamine or banoxantrone, could be utilized to eradicate the hypoxic cell subpopulation (McKeown et al. 2007).

### Future directions

The role of CAIX expression as a biomarker predicting an increased risk of distant metastases needs to be validated in an independent data set. In addition, microarray techniques used in prospective trials will eventually have the potential to identify genomic signatures which correlate with hypoxia, nodal metastasis and overall clinical outcomes. Emerging nuclear medicine PET-imaging techniques, utilizing agents such as F-MISO and Cu-ATSM, may enable non-invasive assessment of hypoxia as a further correlate in the initial staging (Rajendran et al. 2005). It will be important to use these genomic and molecular/physiologic imaging strategies to validate our observation of hypoxia and distant metastasis in early-stage cervix cancer. This tactic would provide a selection criterion for early-stage patients who may benefit from novel adjuvant systemic therapy. Applying these genomic and molecular/physiologic imaging strategies in more advanced stage patients might lead to a better understanding of the mechanisms of action for hypoxia-induced gene therapy, HIF-targeted therapy and anti-angiogenic and anti-vascular therapies.

## Figures and Tables

**Table 1:** Patient and tumor characteristics.

Patients with adequate pathology specimens	118
Patients with recurrence	42
Patients without recurrence	76
Median Follow-Up (months)	59.6
Range	4–195
Median Age (years)	45
Range	19–79
Positive LN at surgery	27
FIGO Stage	
IA	3
IB	105
IIA	7
Unknown	3
Histology	
Squamous Cell Carcinoma	84
Adenocarcinoma	22
Adenosquamous	7
Other	5

**Table 2:** Freedom from recurrence (FFR), freedom from local recurrence as the first site of failure (FFLR) and freedom from distant metastases as the first site of failure (FFDM) as a function of FIGO stage, immunohistochemical score (IHC), pathologic nodal status at surgery and histology on univariate analysis.

Grouping Variable	5-yr Overall FFR	5-yr FFLR	5-yr FFDM
All Eligible Patients	65%		
FIGO Stage:	100% vs 67% vs 13%		
IA vs IB vs IIA	(p = 0.019)		
IHC:	71% vs 62%	73% vs 74%	92% vs 76%
<30 vs ≥30	(p = 0.45)	(p = 0.87)	**(p*=*0.040)**
Lymph Nodes:	40% vs 73%	61% vs 76%	55% vs 90%
Positive vs Negative	**(p = 0.0013)**	(p = 0.18)	**(p < 0.0001)**
Histology:	62% vs 76%	67% vs 90%	83% vs 79%
Squamous vs Other	(p = 0.076)	**(p = 0.0070)**	(p = 0.70)
Age:	64% vs 68%	69% vs 79%	81% vs 83%
<45 vs ≥45 years old	(p = 0.98)	(p = 0.50)	(p = 0.81)

**Table 3:** Freedom from recurrence (FFR), freedom from local recurrence as the first site of failure (FFLR) and freedom from distant metastases as the first site of failure (FFDM) as a function of the combination of immunohistochemical score (IHC) and pathologic lymph node status at surgery.

Grouping Variable	n	5-yr FFR	5-yr FFLR	5-yr FFDM
IHC <30, Negative Lymph Nodes	37	76%	71%	95%
IHC ≥30, Negative Lymph Nodes	54	72%	77%	85%
IHC <30, Positive Lymph Nodes	8	50%	57%	70%
IHC ≥30, Positive Lymph Nodes	19	35%	61%	50%
**P**		**0.0021**	**0.55**	**0.0004**

**Table 4:** Proportional hazard ratios (HR), 95% confidence intervals (CI) and chi-square *P* obtained by fitting survival data to 2- and 3-variable Cox proportional hazard model. The models yield qualitatively similar results when age as a continuous variable or second-order terms (cross-products of the independent variables) are added to the models, though none of these variables approach statistical significance. However, when cross-products are included in the 3-variable model fit to first failure as distant metastases, both IHC (HR **=** 4.99, CI 1.14–61.4, *P* **=** 0.030) and lymph node status (HR **=** 6.76, CI 2.12–25.2, *P* **=** .0015) are significant.

3-Variable Model	Proportional Hazard Ratio (Confidence Interval)		
	p		
	Any Failure	Local Failure	Distant Metastases
IHC:	1.07 (0.57–2.12)	0.81 (0.40–1.70)	2.78 (0.91–12.1)
<30 vs ≥30	p = 0.83	p = 0.58	p = 0.075
Lymph Nodes:	3.24 (1.66–6.16)	2.34 (1.00–5.03)	4.74 (1.83–12.4)
Positive vs Negative	**p = 0.0008**	**p = 0.049**	**p = 0.0016**
Histology:	2.56 (1.22–6.05)	5.37 (1.87–22.7)	1.13 (0.44–3.16)
Squamous vs Other	**p = 0.012**	**p = 0.0008**	p = 0.79

**2-Variable Model**	**Proportional Hazard Ratio (Confidence Interval)**		
	**p**		

	Any Failure	Local Failure	Distant Metastases
IHC:			2.82 (0.57–2.12)
<30 vs ≥30			p = 0.069
Lymph Node Status:	3.28 (1.69–6.18)	2.27 (0.98–4.83)	4.58 (1.83–11.64)
Positive vs Negative	**p = 0.0007**	p = 0.056	**p = 0.0015**
Histology:	2.57 (1.23–6.08)	5.27 (1.83–22.2)	
Squamous vs Other	**p = 0.0108**	**p = 0.0009**	
